# Supplementary material for: Chiral active particles are sensitive reporters to environmental geometry
Source: Nat Commun. 2024 Feb 16;15:1406. doi: 10.1038/s41467-024-45531-5 (PMC10873462; doi:10.1038/s41467-024-45531-5)
Supplement: Supplementary file 1 — Supplementary Information [file 41467_2024_45531_MOESM1_ESM.pdf]

# Supplementary Information for Chiral Active Particles are Sensitive Reporters to Environmental Geometry

Chung Wing Chan[1], Daihui Wu[1], Kaiyao Qiao[1], Kin Long Fong[1,2], Zhiyu Yang[1], Yilong Han[1], Rui Zhang[1*]

[1]Department of Physics, The Hong Kong University of Science and Technology, Clear Water Bay, Kowloon, Hong Kong

[2]Present affiliation: Physik-Department, Technische Universität München, James-Franck-Straße 1, 85748 Garching, Germany

[*]To whom correspondence should be addressed; E-mail: ruizhang@ust.hk.

## Diffusivity of Chiral Active Particles in Free Space

Chiral active particles (CAPs) in free space typically move in a circular path with an orbital radius of $r = v_0/\omega_0$. The long-term diffusivity $D$ can be calculated by the mean squared displacement (MSD) of the trajectory in a two-dimensional space:

$$D = \lim_{t\to\infty} \frac{\langle [\vec{r}(t) - \vec{r}(0)]^2 \rangle}{4t} \tag{S1}$$

In the limit of $t \to \infty$, we can apply Kubo relation (*1*), to express $D$ as follows:

$$D = \frac{1}{2}\int_0^\infty \langle \vec{v}(t)\cdot\vec{v}(t+\tau)\rangle \, d\tau = \frac{v_0^2}{2}\int_0^\infty \langle \cos(\Delta\theta)\rangle \, d\tau \tag{S2}$$

Here, $\langle \cos(\Delta\theta)\rangle$ can be calculated from the solution $P(\theta, t)$ of the Fokker-Plank equation:

$$\frac{\partial}{\partial t} P(\theta, t) = -\omega_0 \frac{\partial P}{\partial \theta} + D_r \frac{\partial^2 P}{\partial t^2} \tag{S3}$$

where $P(\theta, t)$ is the probability for angle $\theta$ during time $t$ (*1*). The explicit form of $P(\theta, t)$ is:

$$P(\theta, t) = \frac{1}{\sqrt{4\pi D t}} e^{-\frac{(\theta - \omega_0 t)^2}{4Dt}} \tag{S4}$$

After integration, we obtain the analytical form of the diffusion constant of CAPs in free space:

$$\begin{aligned} D &= \frac{v_0^2}{2} Re\left[\int_0^\infty \int_{-\infty}^\infty e^{i\theta} P(\theta, \tau) d\theta d\tau\right] \\ &= \frac{v_0^2}{2} \frac{D_r}{D_r^2 + \omega_0^2} \\ &= \frac{v_0^2}{2\omega_0} \frac{\Omega}{1 + \Omega^2} \end{aligned} \tag{S5}$$

with $\Omega = D_r/\omega_0$. When the ratio of the two length scales $\Omega = D_r/\omega_0 = r/l_p = 1$, the diffusivity $D$ of CAPs in free space reaches its maximum value. This ratio characterizes the system as dominated by circular motion (when $\Omega \ll 1$) or noise (when $\Omega \gg 1$). Our simulation results (Fig. S1) are validated with theory, and the measured diffusivity $D$ of CAPs in free space varies across a broad range of $\bar{l}_p \in [10^0, 10^3]$ and $\bar{r} \in [0, 8]$ (Fig. S1d).

## Experimental Setup

The experimental setup is presented in Fig. 1d. Seed particles and 3D-printing particles are placed on a frictional flat cardboard plate glued with an array of circular plastic disks attached on the vibrating stage. The stage vibrates vertically as $A\sin(2\pi ft)$, where $A$ is the amplitude, $f$ is the vibration frequency of the stage, and $t$ is the time. The dimensionless acceleration describes the vibration strength as follows:

$$\Gamma \equiv \frac{a}{g} = \frac{A(2\pi f)^2}{g} \tag{S6}$$

where $a$ is the maximum acceleration of the stage, and $g$ is the free fall acceleration. Two out of the three parameters $A$, $f$, and $a$ are independent (*2*). We set $f = 85$ and $a = 2$ g, which correspond to $A = 0.07$ mm. A SONY A1 camera records the particle motions at 60 frame/s with a Sony FE-50mm-F1.8 lens put vertically 50 cm higher than the plate (Fig. S2) and tracked by OpenCV software for image analysis (*3*). The electromagnetic vibrating stage vibrates the cardboard attached to it via linear bearing to ensure that the vibration is strictly vertical and that the errors of $A$ and $f$ are smaller than $\pm 5$ %.

The orientation of the seed ($\theta_{\text{seed}}$) is directly tracked by image analysis. The velocity direction of the seed is $\theta_{\text{vel}}(t) = \tan^{-1}\left(v_y(t)\,/v_x(t)\right)$, where $v_i$ is measured from the trajectory, $v_i = \left(\vec{r}_i(t+\Delta t) - \vec{r}_i(t)\right)/\Delta t$ for $i = x, y$. The measured $\theta_{\text{seed}} = \theta_{\text{vel}}(t)$ in Fig. S3 shows that the seed always moves along its long axis.

## Kinetic Friction Coefficient Between Seeds and Cardboard Plate

We measure the kinetic friction coefficient $\mu_k$ between seeds and cardboard plates using a 30° inclined plate setup (Fig. S3). The seed slides down without rotation. By capturing the acceleration of the seeds $a_{\text{seed}}$ on the inclined plate via a camera and image analysis, we can calculate

$\mu_k$ by

$$\mu_k = \frac{1}{\sqrt{3}} \left( 1 - \frac{2a_{\text{seed}}}{g} \right), \tag{S7}$$

where $a_{\text{seed}}$ is the acceleration of the seed measured by an image analysis, and $g$ is the gravitational constant. By averaging ten trials of experiment, we find $\mu_k = 0.5$, which is close to the kinetic friction coefficient between two wood (*4*).

## Hopping Motions of CAPs

Particle hopping time $t_{\text{hop}}$ can be calculated as $t_{\text{total}} - t_{\text{slide}}$, representing the total time CAPs spend in hopping between obstacles without contacting surfaces of obstacles. The ratio $\mu$ indicates the dominant mode of motion for CAPs. If $\mu > 1$, it suggests that sliding is the dominant mode of motion. Conversely, if $\mu \leq 1$, it indicates that hopping becomes the dominant mode. Notably, $N$ is proportional to $\mu$. This is because a larger sliding time provides a larger chance for the reversible motion to happen on the surface of obstacles.

We further prepare the histogram of individual hopping time $\delta t$ from leaving an obstacle to landing on another obstacle (Fig. S10). Different peaks in the histograms reflect different collision modes. The position $\delta t$ of each peak corresponds to a hopping arc distance $v_0 \delta t$ (Fig. S9). For example, CAPs with $\bar{r} = 2$ diffusing in the square lattice have a prominent peak at $\delta t \approx 1.5$ (Fig. S10a), indicating that the distance between the two collision sites is about 1.5 (Fig. S9c). The small peak at $\delta t \approx 0.8$ for $\bar{r} = 2$ in Fig. S10a implies a small chance for a short arc distance of $0.8$ between the two collision sites (Fig. S9d). Similarly, for the triangular lattice, the prominent peak at $\delta t \approx 0.9$ with $\bar{r} = 2$ in Fig. S10b implies a popular hopping mode with a collision distance of $0.9$ (Fig. S9e).

Fig. S10 shows that fast and slow diffusions exhibit different large-$\delta t$ tails in their histograms. Fast diffusions (i.e., $\psi > 0$) exhibit fat tails (insets of Fig. S10), that is, more long-distance hopping. By contrast, low-diffusion CAPs primarily undergo short-distance hopping,

in which they become temporarily trapped in local space and are unable to move persistently.

## Irregular Lattices

We have further investigated the imperfect lattice by introducing small spatial variations into the arrangement of square lattice Fig. S8. We choose the packing fraction $\phi = 0.6$ to allow for a wider range of lattice perturbations. To quantify the randomness of the irregular lattice, we measured the spatial variance of the lattice as a disorder parameter by

$$\sigma^2 = \frac{1}{R^2 N} \sum_{N}^{i} (\vec{r}_i - \vec{r}_{0i})^2, \tag{S8}$$

where $\vec{r}_i$ represents the position vector of the obstacle $i$ and $\vec{r}_{0i}$ represents the equilibrium position vector in the corresponding perfect lattice.

The slightly perturbed irregular lattice can be regarded as a combination of different perfect lattices because the CAP dynamics depend on the local density and configuration within the perturbed lattice. We therefore expect that the diffusivity in a perturbed lattice is less radical (compared to the perfect lattice results) and behaves intermediate between the diffusivities in perfect triangular and square lattices of the same packing density. Our experiment and simulation confirm the above expectations (Fig. S8). When a random lattice is well beyond a weakly perturbed lattice, the CAP diffusivity drops below those reported in perfect lattices, as the obstacles lose their spatial correlations and are unable to help the diffusion of CAPs (Fig. S8b). A more thorough study of the effects of randomness on CAP diffusivity is beyond the scope of the current work and we leave it for the future work.

## Topotaxis

We observed a topotaxis effect in a binary lattice consisting of a square lattice on the left and a triangular lattice on the right, both with the identical packing fraction of $\phi = 0.6$. Using a periodic boundary condition for both dimensions, we simulated 500 non-interacting CAPs with

$\bar{r} = 1$ and $\bar{l}_p = 100$. The simulation results demonstrate that the initially randomly placed CAPs tend to migrate from the high-diffusivity triangular region ($D_{\text{eff}} = 0.15$) to the low-diffusivity square region ($D_{\text{eff}} = 0.02$) (Fig. S7). In the experiment, the lack of the periodic boundary condition requires either a long experimental time or many trials, making it difficult to arrive at a clear conclusion. We therefore choose to report on only simulation results regarding this point and leave a systematic study on mixed lattices in the future work.

## Directional Locking Effect with Varying Particle Chirality $\bar{r}$

Simulations show that the reentrant effect is absent in square and triangular lattices when $\bar{r} = 2$ (Fig. S11a, b) for CAPs. We further examine the transition of the directional locking effect crossing $\bar{r} \in [0.5, 2]$ by measuring the strength of direction locking $\epsilon$. Fig. S11c shows that the locking effect weakens non-monotonically as $\bar{r}$ increases. Interestingly, this opposite oscillating trend in square and triangular lattices shows a similar pattern to the opposite diffusive property in square and triangular lattices (Fig. 4c).

## Degree of Lattice Asymmetry

The concept of mirror symmetry inspires us to measure the degree of lattice asymmetry by looking at how much the unit cell of a lattice can overlap with its mirror image. Specifically, to quantify the degree of asymmetry for a parallelogram lattice considered in this work, we measure the maximum possible overlapping area between the unit cell and its mirror (reflected) image along the $45°$ and $90°$ axes. This is achieved by calculating the overlapping areas, denoted as $A_{45}$ and $A_{90}$, which are normalized by the area of the original cell. In particular, $A_{45} = 1$ ($A_{90} = 1$) implies that the image exhibits mirror symmetry along the $45°(90°)$ axes. Smaller values of $A_{45}$ and $A_{45}$ indicate a higher degree of asymmetry in the structure. We further use the greater value of the two to define the maximum overlapping area $A_{\max} = \max(A_{45}, A_{90})$.

## Lattices with Symmetry Breaking

We measure $\Delta D_{\text{eff}}$ as a function of $\delta \in [0, 0.5]$ for $\bar{r} = 1, 1.5$, and $2$ at $\bar{l}_p = 100$. Fig. S12 demonstrates that $\Delta D_{\text{eff}} = 0$ for the three values of $\bar{r}$ when $\delta = 0$ and $0.5$, consistent with our theory that mirror-symmetry-preserving lattices cannot distinguish between clockwise (CW) and counter-clockwise (CCW) particles. The different types of behavior of $\Delta D_{\text{eff}}$ between $\delta = 0$ and $0.5$ are due to particles sensing a different configuration with different chiralities. We focus on $\bar{r} = 1.5$ in our calculations without loss of generality. The result matches the analysis of the overlapping area with a unit cell of the same geometry.

## Difficulty in Directional Locking Experiments

For the direction locking phenomenon, experimental realization is difficult. We tried to apply the external field using gravity. However, the cardboard surface on the inclined plane does not vibrate very uniformly and large noises generated by lifting/tilting the vibration table, which can cause damage to the vibration system. Moreover, vibrating inclined plane can rotate the light seed which is beyond our simulation model. We managed to perform a few trials within a short period, while the directional locking effect is not observed in experiment due to the above problems. With these experimental difficulties, we leave it for the future work.

## Wiener Process

Wiener process is a standard mathematical model for Brownian motion and is represented by a random variable $W(t)$ over a time span $[0, T]$ such that (*2*):

- $W(0) = 0$;
- For $0 \leq t_1 < t_2 \leq T$, the random variable $W(t_2) - W(t_1)$ obeys the distribution $\mathcal{N}(0, t_2 - t_1)$;

- For $0 \leq t_1 < t_2 < t_3 < t_4 \leq T$, the random variables $W(t_2) - W(t_1)$ and $W(t_4) - W(t_3)$ are independent.

- For $0 \leq t_1 < t_2 < t_3 < t_4 \leq T$, the random variables $W(t_2) - W(t_1)$ and $W(t_4) - W(t_3)$ are independent.

Where $\mathcal{N}(0, t_2 - t_1)$ is a normal distribution with zero mean and standard deviation $t_2 - t_1$. Considering that a sample from the Wiener process takes a random value for every $t \in [0, \infty)$, we specify the times $t_1 < t_2 < ... < t_N$ for each $W(t)$, and:

$$\Delta W(t_n) = W(t_{n+1}) - W(t_n),$$

where $W(t_0) = W(0) = 0$, then $\Delta W(t_n) = \mathcal{N}(0, t_{n+1} - t_n) = \sqrt{\Delta t}\mathcal{N}(0, 1)$ and $\Delta t = t_{n+1} - t_n$. We can, therefore, sample all the values of the $\Delta W(t_n)$ independently.

## Euler–Maruyama Method

Euler–Maruyama method is the analogy of Euler method for deterministic integrals applied to the stochastic case. Given a scalar stochastic differential equation with drift and diffusion constant $\omega_0$ and $\sqrt{2D_r}$:

$$d\theta(t) = \omega_0 dt + \sqrt{2D_r}\xi(t)dt, \tag{S9}$$

where $\xi(t)$ is a random noise that can be modeled by Wiener process $\xi(t)dt = dW(t)$. Euler-Maruyama method approximates $\theta(t)$ by

$$\begin{aligned} \theta(t_{n+1}) &= \theta(t_n) + \omega_0 \Delta t + \sqrt{2D_r}\Delta W(t_n) \\ &= \theta(t_n) + \omega_0 \Delta t + \sqrt{2D_r \Delta t}\mathcal{N}(0, 1), \end{aligned} \tag{S10}$$

where $\Delta t > 0$ is the time step.

## Simulation Method

We simulate the particle trajectories by numerically integrating Eq. 1 with Euler–Maruyama (*5, 6*) method. If the update step moves the particle into obstacles, then the particle–obstacle interaction force (Eq. 3) is triggered. The normal component of the attempted displacement is removed. The actual displacement is given by the tangential component of the attempted displacement $\vec{r}_n = \vec{r}_{n-1} = \vec{r}_{n-1} + v_0 \Delta t [\hat{p} \cdot \hat{T}] \hat{T}$, where $\hat{T}$ is the tangential unit vector of the obstacle surface at the point of the surface closest to $r_{n-1}$. In the above simulations, we fix $v_0 = R = 1$ and vary $D_r$ to control $\bar{l}_p$. We choose simulation time step $\Delta t$, which is much smaller than $\tau_p$, such that $(v_0 + v_g)\,\Delta t \ll R$.

# References


1. L. Schimansky-Geier, U. Erdmann, N. Komin, *Physica A: Statistical Mechanics and its Applications* **351**, 51 (2005).

2. L. Guan, L. Tian, M. Hou, Y. Han, *Scientific Reports* **11**, 16561 (2021).

3. G. Bradski, *Dr.Dobb's Journal of Software Tools* (2000).

4. R. T. Barrett, Fastener design manual, *Tech. rep.* (1990).

5. M. Balvin, E. Sohn, T. Iracki, G. Drazer, J. Frechette, *Physical Review Letters* **103**, 078301 (2009).

6. E. A. Novikova, M. Raab, D. E. Discher, C. Storm, *Physical Review Letters* **118**, 078103 (2017).

# SUPPLEMENTARY FIGURES

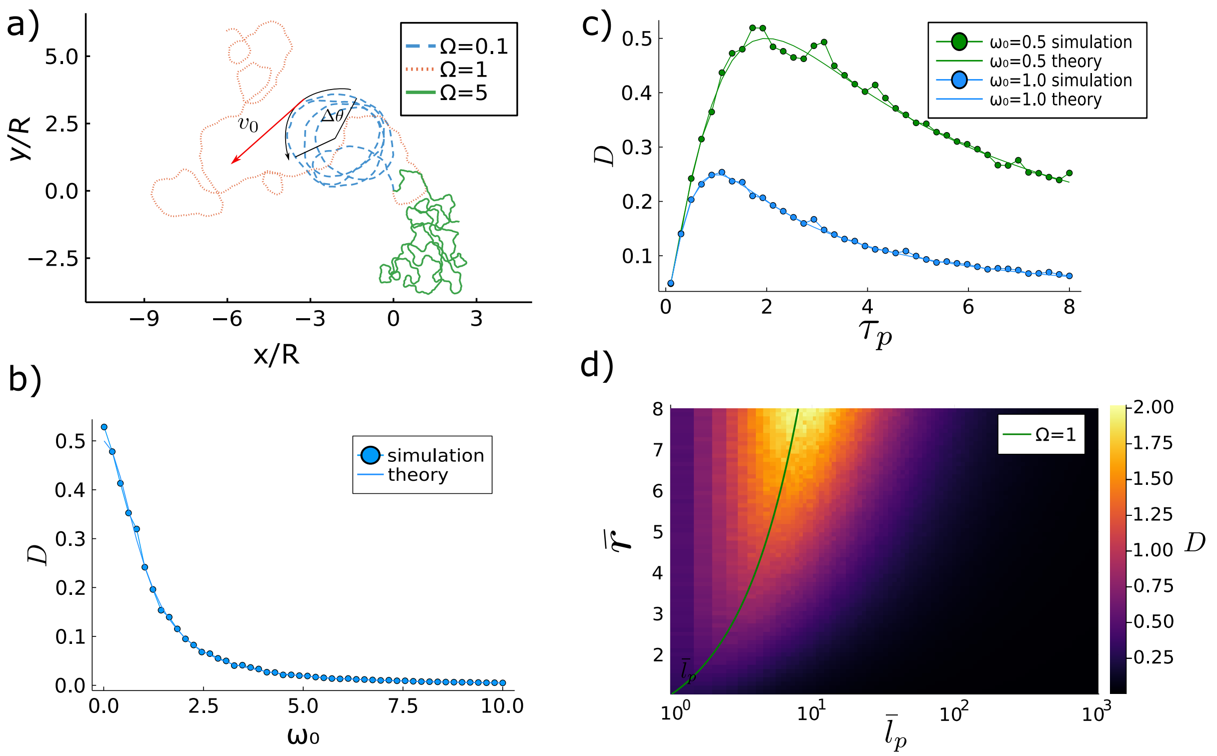


Figure S1: **Diffusivity $D$ and trajectories of CAPs in free space.** (a) Trajectories of CAPs in free space with $\Omega = 0.1,\ 1,$ and $0.5$. (b–c) Simulation results fitted with Eq. S5. (b) $D$ as a function of angular velocity $\omega_0$ while keeping $v_0 = 1$ and $D_r = 1$. (c) $D$ as a function of persistent time $\tau_p$ for $\omega_0 = 0.5$ (green) and $\omega_0 = 1$ (blue) while keeping $v_0 = 1$. (d) $D$ of CAPs in free space in $\bar{l}_p \in [10^0, 10^3]$ and $\bar{r} \in [0, 8]$.

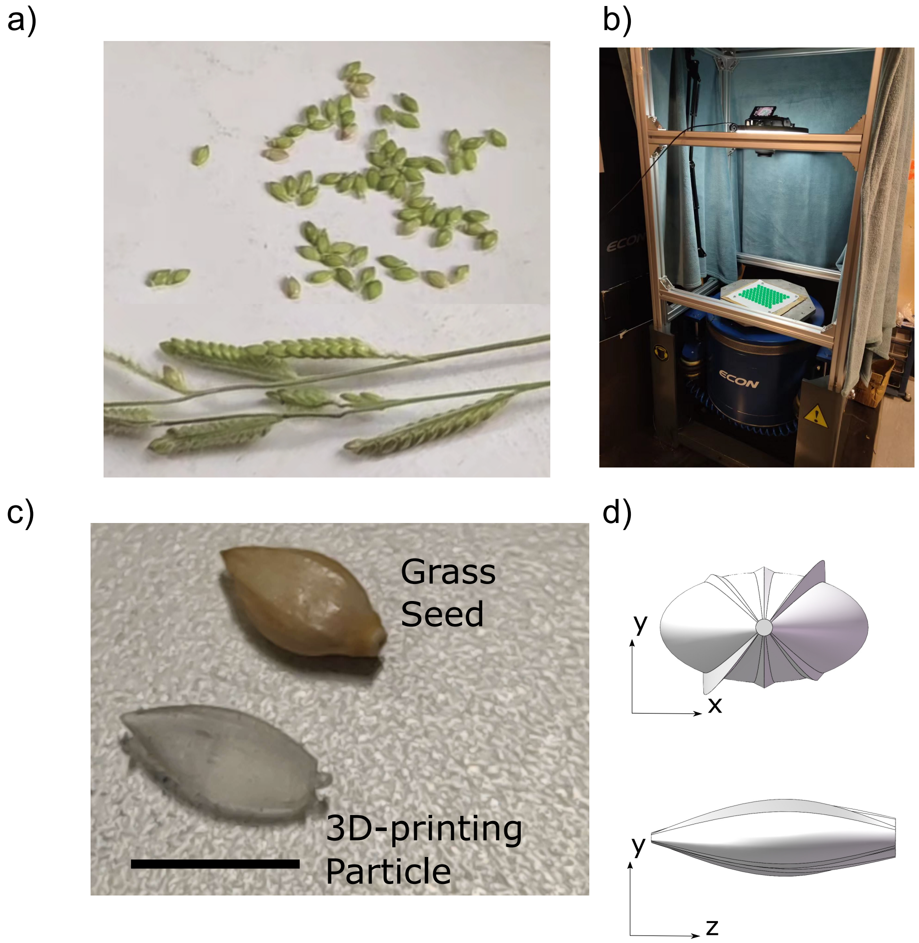


Figure S2: **Experimental system.** (a) Grass and its seed. (b) Experimental setup corresponding to Fig. 1 in the main text. (c) Grass seed and 3D printing particle. Scale bar: 3 mm. (d) Side views of the design of the 3D-printing particle.

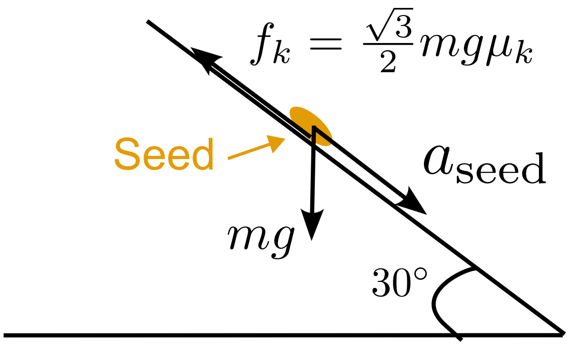


Figure S3: **Measurement of friction coefficient.** The free-body diagram of a seed siding along a 30° inclined plane.

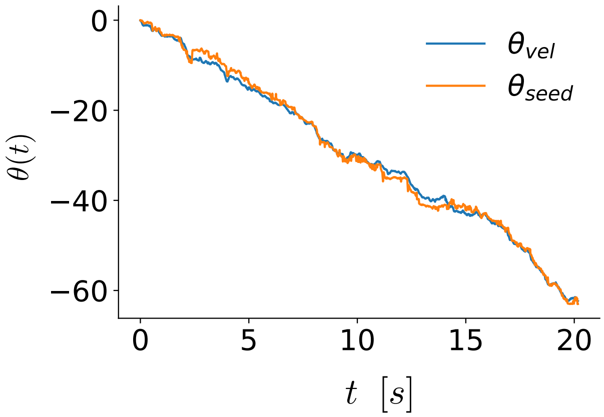


Figure S4: **Seed orientation and velocity direction.** Velocity direction of the seed along its long axis.

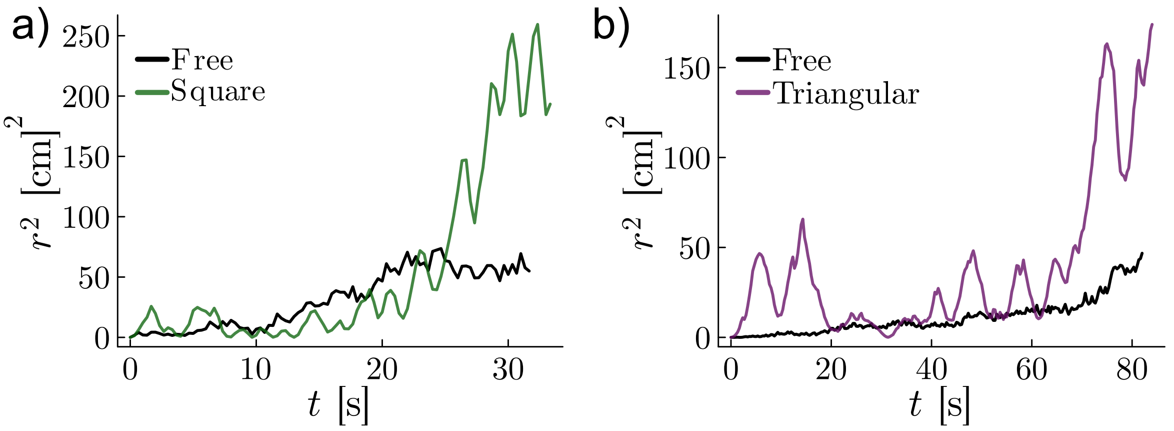


Figure S5: **Square displacements (SD)**. (a) SDs of Seed 1 in a square lattice and free space. (b) SDs of Seed 2 in a triangular lattice and free space.

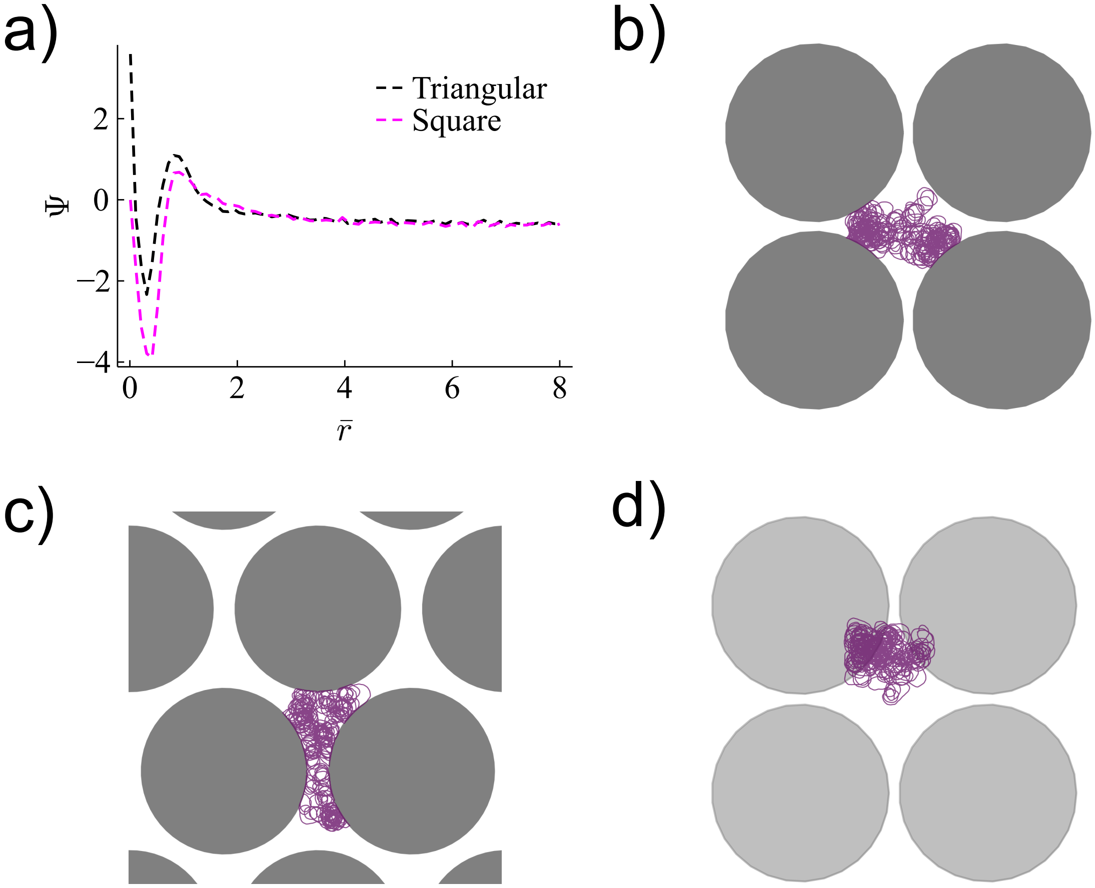


Figure S6: **CAPs in low $\bar{l}_p$ regime.** (a) Logarithmic ratio of particle's diffusivity in an obstacle lattice to that in free space $\Psi = \ln \frac{D\text{eff}}{D}$ at $\bar{l}_p = 1$. (b–c) The trajectory of CAPs at $\bar{l}_p = 1$ and $\bar{r} = 0.1$ in square (b) and triangular (c) lattices. (d) Trajectory of CAPs in free space. Circles are not obstacles and are used for comparison only.

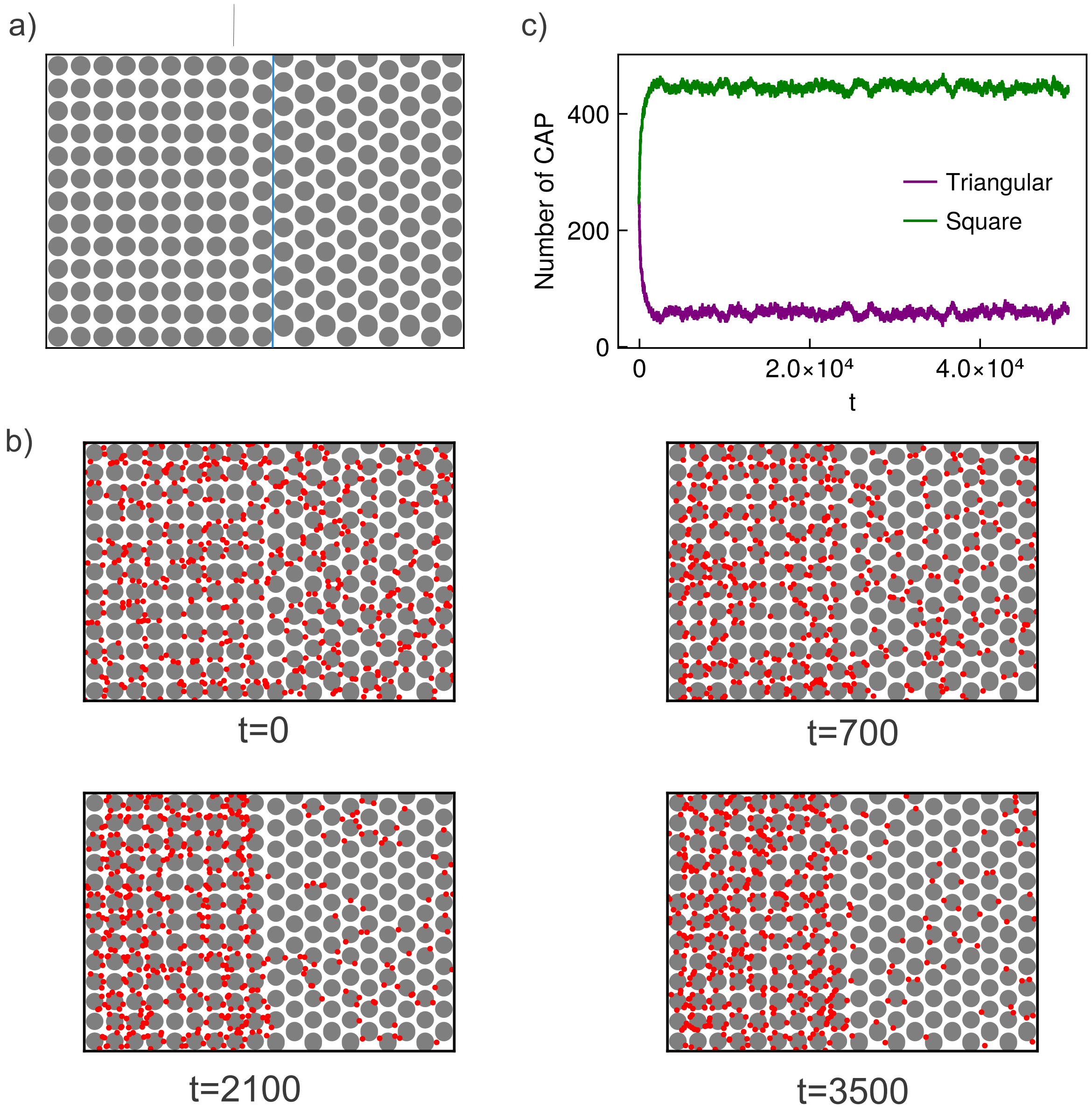


Figure S7: **Topotaxis in a binary lattice.** (a) The square lattice on the left and the triangular lattice on the right with $\bar{r} = 1$ and $\bar{l}_p = 100$ and the same packing fraction $\phi = 0.6$. The blue line indicates their boundary. (b) Snapshots of 500 non-interacting CAPs (red dot) show a topotaxis effect, i.e., migrating from the high-diffusivity triangular region ($D_{\text{eff}} = 0.15$) to the low-diffusivity square region ($D_{\text{eff}} = 0.02$). Particles are randomly placed at $t = 0$. (c) The evolution of the number of CAPs in the triangular (purple) and square (green) lattices.

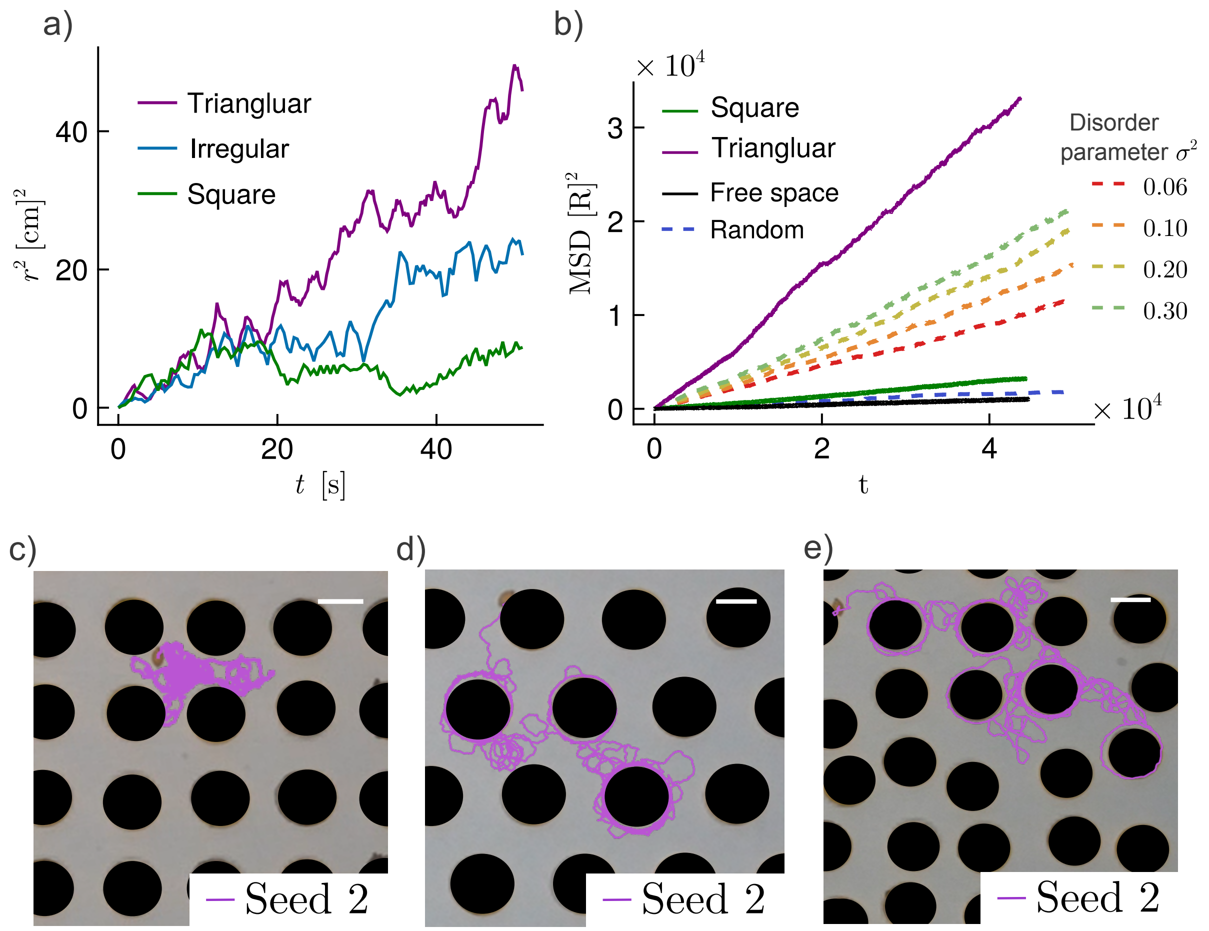


Figure S8: **CAP diffusion in ordered and disordered lattices with $\phi = 0.6$.** (a) Experimental MSDs in ordered (c and d) and disordered (e) lattices. (b) Simulated MSDs for CAPs with $\bar{r} = 1$ and $\bar{l}_p = 100$ in different obstacle lattices. The irregular lattices used in (a) has variation 0.2. The diffusivity deviates from that in a square lattice and approaches that in a triangular lattice as the degree of disorder $\sigma^2$ increases. (c–e) Trajectories of seed 2 in ordered and disordered obstacle arrays for a duration of 60 s. $\sigma^2 = 0.2$ in (e). The trajectory in the irregular lattice exhibits caging in the square lattice and fast diffusion in the triangular lattice. Scale bars: 1 cm.

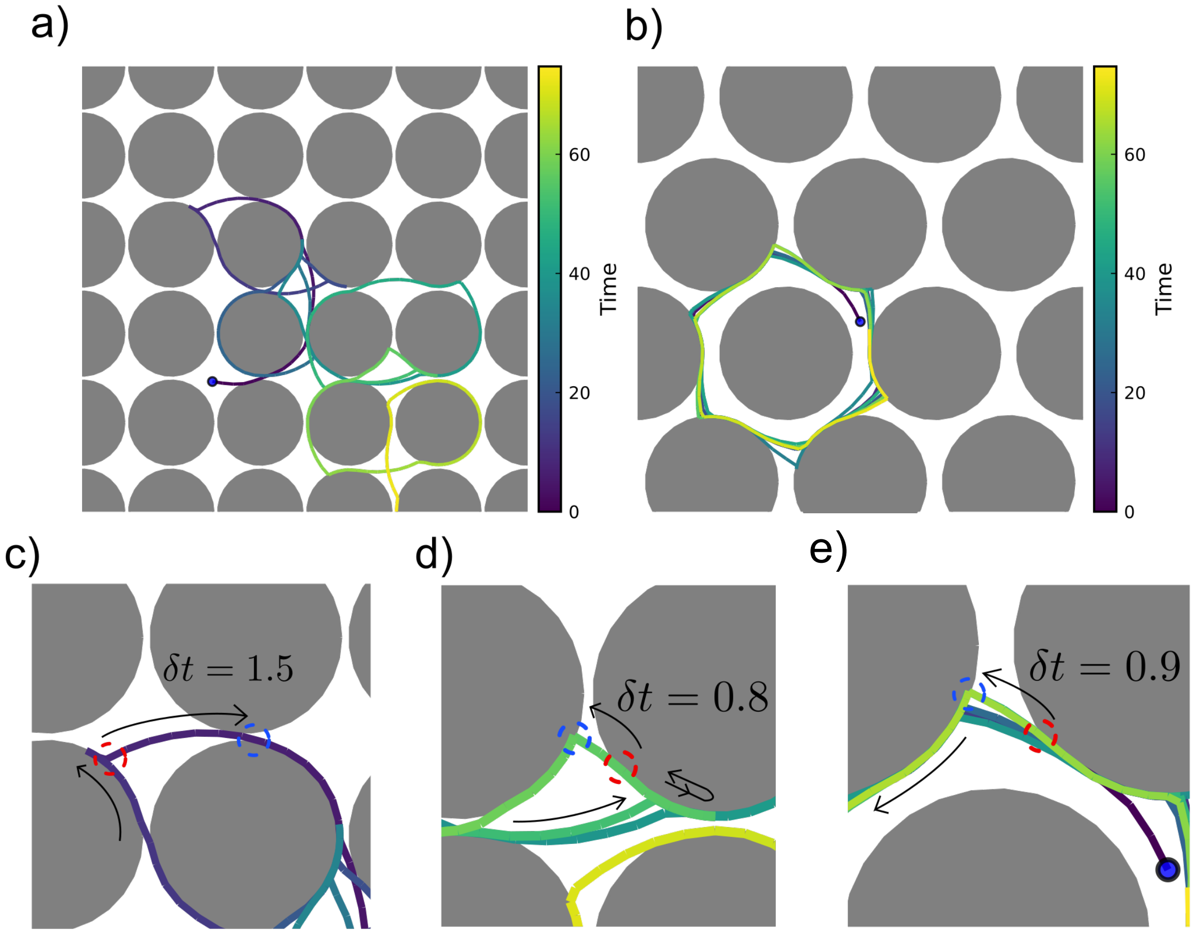


Figure S9: **Different collision modes at $\bar{r} = 2$.** (a) Typical trajectory of CAP with $\bar{r} = 2$ in square lattice. (b) At special magic values (e.g. $\bar{r} = 2$ and $\phi = 0.7$), the CAP trajectory in the triangular lattice is nearly closed. (c–e) Zoom-in of (a-b) shows three collision modes with hopping time $\delta t = 1.5$ (c), $\delta t = 0.8$ (d) in the square lattice, and $\delta t = 0.9$ (e) in the triangular lattice. Black arrows represent the velocities. Red and blue dashed circles represent the launching and landing sites, respectively.

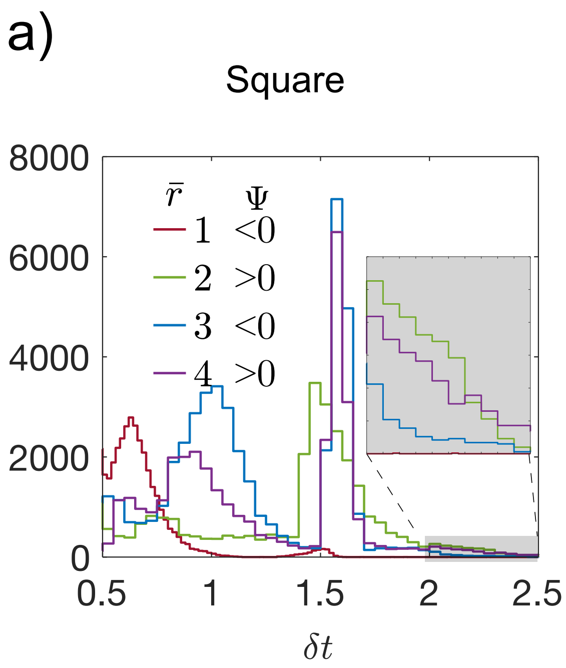


Figure S10: **Hopping motions of CAPs.** (a, b) Histograms of hopping time interval $\delta t$ of square (a) and triangular (b) lattices. Insets show fast diffusive CAPs exhibiting fat large-$\delta t$ tails in their histograms.

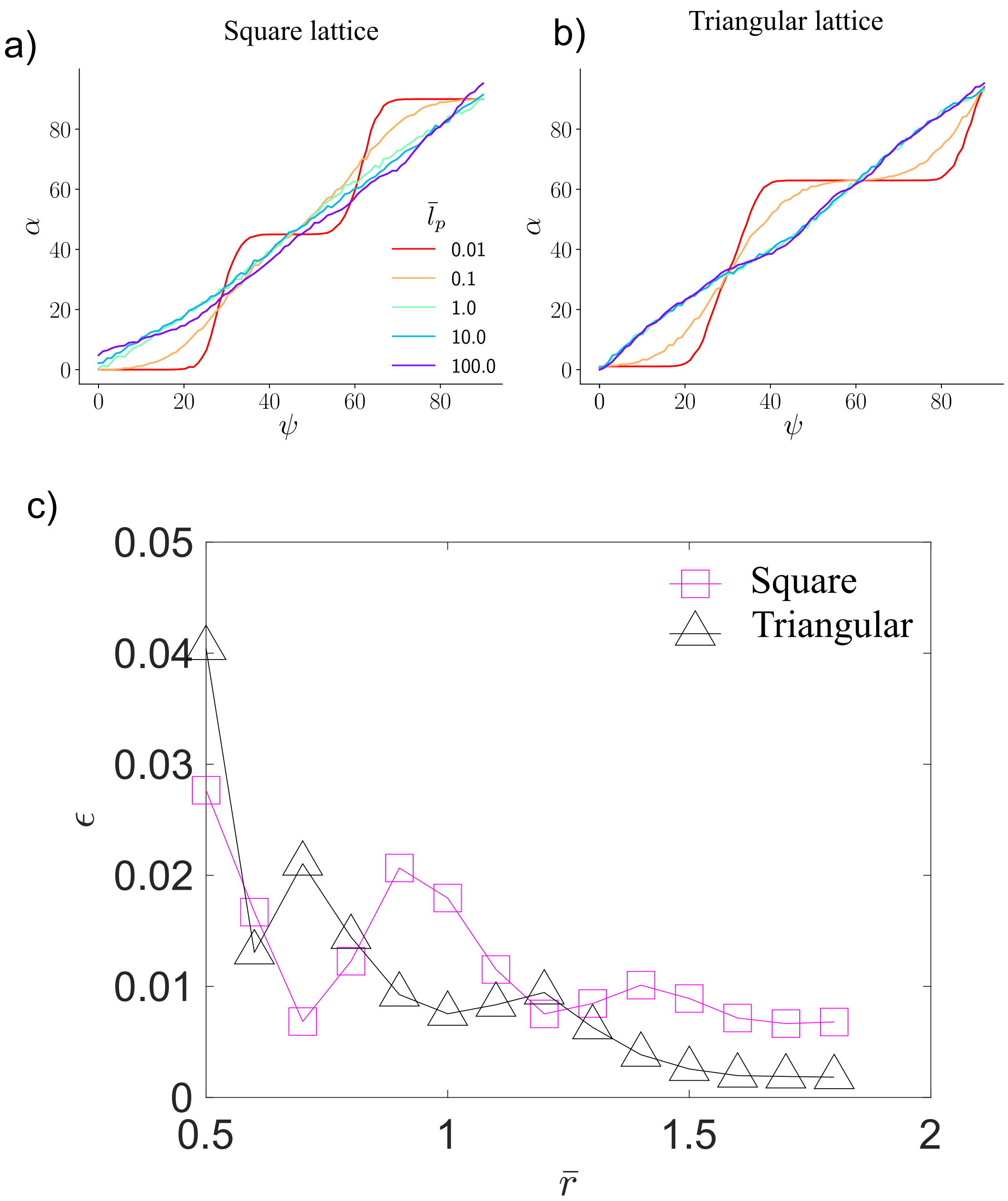


Figure S11: **Directional locking effect for different $\bar{r}$.** (a, b) Directional locking effect in the square (a) and triangular (b) lattices at $\bar{r} = 2$ and different $\bar{l}_p \in [0.001, 0.1, 1, 10, 100]$. (c) Strength of direction locking $\epsilon$ in the square lattice and triangular lattice with $\bar{l}_p = 100$ and $\bar{r} \in [0.5, 2]$.

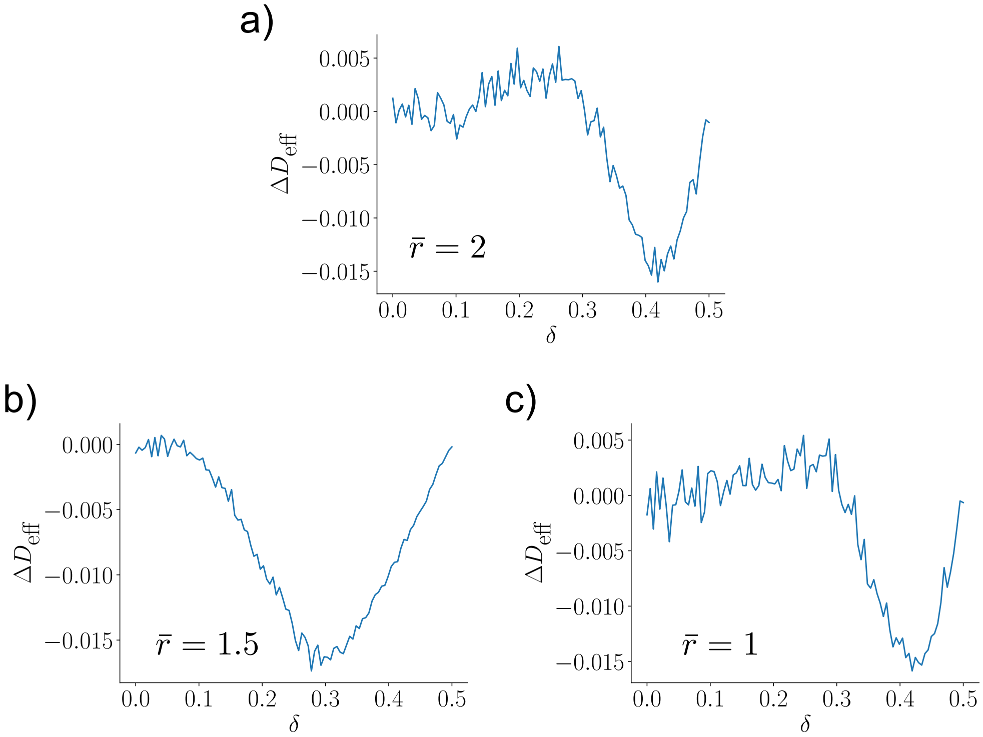


Figure S12: **$\Delta D_{\mathbf{eff}}$ in parallelogram lattices.** Difference in effective diffusivity between CW and CCW particles $\Delta D_{\mathrm{eff}}$ in parallelogram lattices with $\bar{r} = 2$ (a), $1.5$ (b), and $1$ (c) in $\bar{l}_p = 100$ and $\delta \in [0, 0.5]$.
