# Supplementary material for: Chiral active particles are sensitive reporters to environmental geometry
Source: Nat Commun. 2024 Feb 16;15:1406. doi: 10.1038/s41467-024-45531-5 (PMC10873462; doi:10.1038/s41467-024-45531-5)
Supplement: Supplementary file 3 — Description of Additional Supplementary Video Flies [file 41467_2024_45531_MOESM3_ESM.docx]

File Name: Supplementary Video 1

Description: Seed 1 self-propelling in free space on the vibration stage.

File Name: Supplementary Video 2

Description: Seed 2 self-propelling in free space on the vibration stage.

File Name: Supplementary Video 3

Description: 3D-printing particles on the vibration stage.

File Name: Supplementary Video 4

Description: Seed 1 self-propelling in a square lattice exhibiting fast diffusion.

File Name: Supplementary Video 5

Description: Seed 2 self-propelling in a square lattice exhibiting slow diffusion.

File Name: Supplementary Video 6

Description: Seed 1 self-propelling in a triangular lattice exhibiting slow diffusion.

File Name: Supplementary Video 7

Description: Seed 2 self-propelling in a triangular lattice exhibiting fast diffusion.

File Name: Supplementary Video 8

Description: A CW CAP (seed 1) self-propelling in a CCW parallelogram lattice with $\delta=-0.315$ exhibiting fast diffusion.

File Name: Supplementary Video 9

Description: A CW CAP (seed 1) self-propelling in a CW parallelogram lattice with $\delta=0.315$exhibiting slow diffusion.

File Name: Supplementary Video 10

Description: Overlapping area along the $45^{\circ}$ axis.

File Name: Supplementary Video 11

Description: Overlapping area along the $90^{\circ}$ axis.
